# Evidence of Maternal Offloading of Organic Contaminants in White Sharks (*Carcharodon carcharias*)

**DOI:** 10.1371/journal.pone.0062886

**Published:** 2013-04-30

**Authors:** Christopher G. Mull, Kady Lyons, Mary E. Blasius, Chuck Winkler, John B. O’Sullivan, Christopher G. Lowe

**Affiliations:** 1 Department of Biological Sciences, California State University Long Beach, Long Beach, California, United States of America; 2 Institute for Integrated Research in Material Environments and Society, California State University Long Beach, Long Beach, California, United States of America; 3 Southern California Marine Institute, Terminal Island, California, United States of America; 4 Monterey Bay Aquarium, Monterey, California, United States of America; Federal University of Rio de Janeiro, Brazil

## Abstract

Organic contaminants were measured in young of the year (YOY) white sharks (*Carcharodon carcharias*) incidentally caught in southern California between 2005 and 2012 (n = 20) and were found to be unexpectedly high considering the young age and dietary preferences of young white sharks, suggesting these levels may be due to exposure *in utero*. To assess the potential contributions of dietary exposure to the observed levels, a five-parameter bioaccumulation model was used to estimate the total loads a newborn shark would potentially accumulate in one year from consuming contaminated prey from southern California. Maximum simulated dietary accumulation of DDTs and PCBs were 25.1 and 4.73 µg/g wet weight (ww) liver, respectively. Observed ΣDDT and ΣPCB concentrations (95±91 µg/g and 16±10 µg/g ww, respectively) in a majority of YOY sharks were substantially higher than the model predictions suggesting an additional source of contaminant exposure beyond foraging. Maternal offloading of organic contaminants during reproduction has been noted in other apex predators, but this is the first evidence of transfer in a matrotrophic shark. While there are signs of white shark population recovery in the eastern Pacific, the long-term physiological and population level consequences of biomagnification and maternal offloading of environmental contaminants in white sharks is unclear.

## Introduction

Marine pollutants, such as persistent organic pollutants (e.g. DDT, PCBs), are a global concern for ecosystems, wildlife, and human health. The majority of pollutants released into the environment ultimately end up in aquatic ecosystems, where they can remain in sediments or be assimilated into food webs and reach potentially harmful levels in higher trophic level organisms or humans [1]. Of particular concern are marine ecosystems adjacent to heavily populated or industrialized areas that have elevated levels of organochlorine contaminants such, as DDT and PCBs [1]. In southern California, an estimated 110 tons of ΣDDT and 11 tons of ΣPCBs remain in marine sediments at historic dumping sites along the Palos Verdes Peninsula, despite prohibition of discharging these organic pollutants in the 1970s [2], [3]. The persistently high concentrations of contaminants at the Palos Verdes Peninsula and their ongoing redistribution due to physical perturbation and biological processes pose health concerns for marine organisms utilizing southern California, especially higher trophic level predators [4], [5].

Due to their nonpolar nature, DDT and PCBs tend to be taken up by living organisms, and eventually are biomagnified throughout the food web. Marine apex predators are particularly susceptible to accumulating high levels of these contaminants because of their trophic position, longevity, and inability to process and excrete many of these anthropogenic compounds [6], [7], [8], [9]. Elevated levels of DDT and PCBs have been observed in polar bears, *Ursus maritimus* [10], [11], pinnipeds [12], and odontocetes [13], [14], which are top marine predators in their respective communities. While many sharks provide similar ecosystem functions as other top predators [15] much less is known about the levels and effects of these contaminants on this group. However, the few studies that have been conducted on elasmobranch fishes indicate that they bioaccumulate organic compounds readily due to their higher trophic position, life history characteristics (i.e. slow growth, longevity), and large, lipid rich livers where contaminants can be accumulated [16], [17], [18], [19], [20].

White sharks (*Carcharodon carcharias*) share similar ecological roles and reproductive life-history traits with the better-studied marine mammals, in that both nourish offspring through the metabolism of stored maternal lipids (i.e. liver and blubber respectively), which is also the primary site of organic contaminant accumulation. Lipids are mobilized from blubber during milk production in mammals and from the liver during vitellogenesis in sharks, then subsequently passed to offspring during lactation or oophagy (developing embryos feed on sequentially ovulated unfertilized eggs). These pathways provide a mechanism for the passive transfer of bioaccumulated contaminants from mother to offspring during lactation or gestation. This offloading of contaminants to offspring has been well documented in several taxa besides marine mammals [21], [22], [23], [24], including birds [25], [26], [27], reptiles [28], [29], [30], and teleost fishes [31], [32], [33]. However despite their global distribution, and evidence of bioaccumulation of organochlorines in sharks [34], little attention has been paid to maternal offloading processes. Evidence of maternal transfer of contaminants, specifically detectable levels of ΣDDT, was noted in a single study of Atlantic spiny dogfish (*Squalus acanthias*) [35], a relatively long lived species capable of considerable bioaccumulation. Elevated levels of ∑DDT in the livers of neonatal sharks were attributed to transfer via yolk during embryonic provisioning. However, since Atlantic spiny dogfish are not apex predators, and are lecithotrophic (e.g. the developing embryo is nourished solely by the initial yolk-sac), it may not be indicative of the greatest potential of maternal offloading in sharks. Thus, the dramatic effects of biomagnification of contaminants and subsequent transfer to offspring would be difficult to compare to other well-studied systems such as marine mammals.

White sharks are the most recognizable marine apex predators of temperate and subtropical oceans. Southern California and Baja California, Mexico are known nursery areas for northeastern Pacific white sharks, where young of the year (YOY,<one yr. old) and juvenile sharks occur close to shore during summer and fall [36], [37], [38], [39]. Extremely high levels of DDT and PCBs have been observed in YOY white sharks from southern California [16], which was unexpected considering their young age. Despite the legacy of contamination in southern California, the levels of contaminants observed in these YOY white sharks [16] are not likely to have been achieved by dietary exposure alone, though this has not been directly tested until now. Therefore, maternal offloading may help explain these elevated contaminant concentrations with regards to adult white sharks’ high trophic position and oophagous method of matrotrophy [40].

To estimate the potential contribution of maternally derived contaminants to observed levels in YOY white sharks from southern California, we developed a bioaccumulation model to simulate contaminant uptake through diet alone. The model was designed to estimate the maximum possible amount of dietary bioaccumulation in YOY white sharks using contaminant levels of known prey found in southern California. We model bioaccumulation of contaminants from dietary exposure and compare with the results of Mull et al. [16], with additional samples from YOY white sharks to assess the role of maternal offloading in elevated contaminant concentrations. We hypothesized that YOY white sharks would exhibit contaminant loads exceeding those of our model output, indicating that the observed levels cannot be achieved via feeding alone, and that maternal offloading is an important factor explaining the high observed levels of organochlorine contaminants.

## Materials and Methods

### Sample Collection

**Figure 1:**
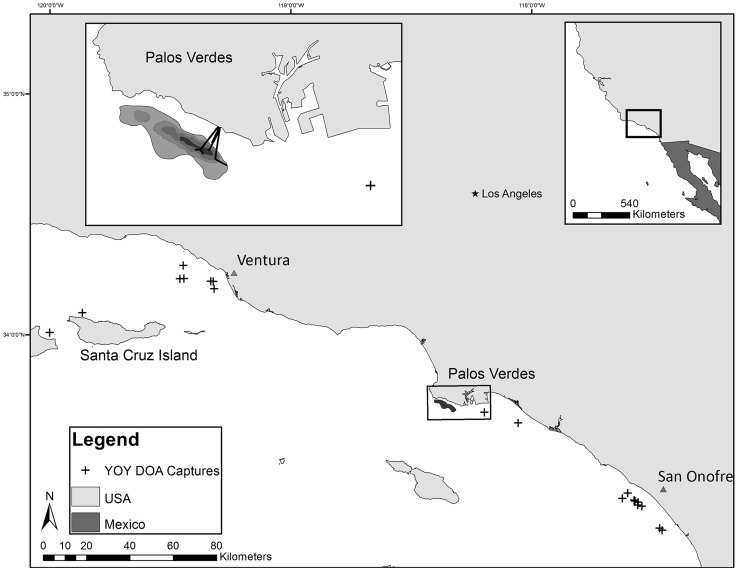
Map of the study area in the Southern California Bight (SCB). Black crosses denote the capture location of each individual used in this study. The shaded area represents the Palos Verdes Shelf Superfund Site where large amounts of DDT and PCBs were discharged with effluent and constitute a large portion of the legacy contaminants in the SCB.

Juvenile white sharks are occasionally caught incidentally in several southern California fisheries [39] (Figure 1). Deceased animals were collected and brought back to the CSULB Shark Lab for dissection and tissue collection. Information about sex, total length (TL), weight and date of capture was recorded (Table 1); no animals were killed for the purposes of this study. Sharks were considered YOY if they measured under 175 cm TL [41]. Approximately 20 g of liver was collected from the middle of the left lobe from each shark, wrapped in foil for later organochlorine analyses, and stored in a freezer at −20°C until extractions could take place (n = 20).

**Table 1:** Capture date, total length (TL), percent lipid concentration of the liver and liver concentrations (µg/g, wet weight) for ΣDDT and ΣPCB for the YOY white shark samples.

Capture Date	TL (cm)	Sex	% Lipid	ΣDDT	ΣPCB
27-Jul-10	116	F	59	51.3	10.0
26-Sep-12	130	M	63	287.0	38.2
28-Aug-09	136	M	37	4.2	1.3
16-Oct-08	137	F	75	60.2	17.7
23-Aug-07	140	M	62	37.1	15.0
31-Jul-12	140	F	75	45.6	10.3
7-Aug-05	141	F	65	186.0	28.3
13-Jun-06	143	M	77	18.4	10.5
12-Jul-12	148	F	60	136.2	21.6
9-Aug-08	148	F	79	58.4	13.1
23-Aug-12	150	M	58	110.3	21.6
26-Aug-07	151	M	62	21.9	7.3
4-Aug-09	156	F	62	34.9	14.7
16-Aug-10	156	F	70	177.9	26.7
1-Oct-09	158	F	51	74.5	21.4
13-Jun-10	158	M	38	249.2	24.9
26-Jun-11	160	M	84	276.2	27.6
6-Jul-10	166	F	79	19.4	2.9
17-Aug-08	167	F	78	33.0	10.8
17-Apr-09	175	F	26	26.2	4.7
Mean			63	95.4	16.4
SD			15.5	91.4	9.7

#### Sample analysis

Samples of liver were analyzed for DDT and its metabolites (herein DDT) and PCBs using gas chromatography mass spectrometry (GCMS). Contaminant data for sharks caught between 2005–2009 was obtained from previously published study [16] and sample analysis for sharks caught between 2010–2012 is described below. In both cases, analytical procedures are nearly identical.

Prior to analysis, liver tissue samples were passed through several preparative steps to extract, purify, and concentrate the contaminants from the neutral lipids. Due to equipment accessibility at IIRMES, two extraction methods (microwave and Soxhlet) were employed. Samples prior to 2010 were extracted using a MARS 5 microwave reaction system (CEM Corporation, Matthews, NC) while all samples taken after 2009 underwent Soxhlet extraction. All post-extraction procedures were identical. Despite the difference in extraction method, all batches passed QA/QC criteria (described below), and we are therefore confident these methods are comparable. Prior to extraction all samples were spiked with recovery surrogates to determine the extraction efficiency and retention of compounds through the preparatory process. A 1–2 g subsample of liver was used in both procedures and were extracted with a 3∶1 mixture of dichloromethane (DCM):acetone. For all samples, lipid content was determined gravimetrically from split aliquots of the extracts after removing the DCM [42].

After extraction, the remaining extracts were processed using Alumina-B/Silica Gel chromatography by sequential elution with hexane, 30% DCM in n-hexane, and DCM. The samples were then concentrated by rotavap, transferred to an autosampler vial, and internal standards (4,4′-Dibromobiphenyl and 2,2′,5,5′-Tetrabromobiphenyl) were added prior to chemical analysis. Samples were injected onto an Agilent gas chromatograph (GC; 6890N series) equipped with a mass selective detector (MSD; Agilent 5973 inert series) using an autosampler (7683B series, Agilent Technologies, Santa Clara, California, USA). The GC column used was a ZB-5 (J&W Scientific; Santa Clara, California) fused silica capillary (0.25 mm ID×60 m) with 0.25 µm film thickness. The temperature profile of the GC oven was programmed from 45°C to 125°C at 20°C/min, then to 295°C at 2.5°C/min and held for 10 min. Injector and transfer line temperatures were set at 285°C and 300°C, respectively. The source and quadrupole temperatures were set at 230°C and 150°C, respectively. Helium was used as the carrier gas at a flow velocity of 40 cm/sec. The MSD was used in the Electron Ionization (EI) mode and scanned from 45–500 amu at a rate of 1.66 scans/sec. Data was acquired by software in the GCMS system. Total PCB (∑PCB) concentrations was calculated as the sum of 53 individually resolved peaks of PCBs congeners: 3 8,18, 28, 31, 33, 52, 49, 44, 37, 74, 70, 66, 95, 56, 101, 99, 119, 87, 97, 81, 110, 77, 151, 123, 149, 118, 114, 153, 168+132, 105, 105, 141, 138, 158, 126, 187, 183, 128, 167, 174, 177, 156, 180, 169, 170, 201, 189, 195, 194, 206, and 209. Total DDT (∑DDT) was calculated as the sum of 2,4′-DDE, 4,4′-DDE, 2,4′-DDD, 4,4′-DDD, 2,4′-DDT and 4,4′-DDT. Quantification of each target analyte was based on the largest single ion with confirmation from at least two additional ions [43].

### Quality Assurance/Quality Control (QA/QC)

For quality assurance and quality control, method blanks (n = 5), duplicates (n = 4), matrix spikes (MS)/matrix spike duplicates (MSD), and standard reference materials (SRMs; n = 4) were processed in parallel with samples. All target analytes in the laboratory blanks were below detectable concentrations. Precision of the method was evaluated by one duplicate analysis of shark liver per 5 samples analyzed; the relative difference for PCBs and DDT of the four duplicates was within 14% and 9% of each other. A known concentration of the target analytes (160 ng and 250 ng for PCBs and DDT, respectively) was spiked into the sample matrix as a check for matrix effects through the calculation of recoveries. Matrix spike recoveries of the target analytes were within an acceptable range of 70 to 130% (EPA Method 8000), with a relative significant difference (RSD) of <10%. For PCBs, MS/MSD percent recoveries (mean ± SD) were 75±10% and 79±12%, respectively with a RSD of 5%. For DDT, MS/MSD percent recoveries were 71±14% and 77±23%, respectively with a RSD of 6%. Lake Michigan fish tissue SRM (1947; National Institute of Standards and Technology) was within the acceptable percent recovery limit (±30%). Recovery of PCBs and DDT recovery surrogates were 104±12% and 82±12%, respectively. Four recovery surrogates (tetrachloro-m-xylene, TCMX; PCB 30, PCB 112 and PCB 198) were added to each sample prior to extraction to follow analyte recovery. Liver sample recoveries (n = 20; mean ± SD) of TCMX, PCB 30, PCB 112 and PCB 198 were 114±23%, 110±20%, 106±24%, and 78±9%, respectively. Although overall MS/MSD recovery was low, recovery of the most abundant analytes was very high (e.g. 4,4′-DDE which represent 99% of DDT had a recovery of 106.5%). The recovery of abundant analytes in addition to other QA/QC criteria gives us high confidence in the reported values.

### Data Analysis

No corrections were made to sample values based on recovery factors because they were all in the acceptable range (70–130%). Values for duplicate samples analyzed were averaged for data summaries and statistical analyses. Values below detection limits were treated as zero values. All organic analyte values for samples were expressed on a wet-weight basis (µg of analyte per g wet weight tissue). All analyses were carried out using Sigmaplot 11.0 (Systat Software Inc., San Jose, CA) and the R statistical package [44].

A potential confounding factor in interpreting tissue contaminant concentrations is the effect of body condition. Poor body condition, characterized by low body mass or low body mass of lipid storage tissues, can artificially increase the concentration of contaminants. In pinnipeds body condition is inversely correlated with contaminant concentrations, especially post-weaning when lipid reserves are heavily relied upon prior to efficient foraging [45], [46]. During the first few weeks of life neonatal sharks rely heavily on lipid reserves in livers [47], [48], [49]. This reliance on liver reserves during early life could increase organochlorine contaminant concentrations in young sharks as lipids are metabolized but contaminants are not. To ensure that our results were not confounded by this phenomenon we tested for correlations between organochlorine concentrations and body conditions, measured as hepatosomatic index (liver mass:total body mass) and lipid content (%) of livers. Contaminant levels were log10 transformed and checked with Shapiro-Wilk test to ensure normality.

### Bioaccumulation Modeling

Bioaccumulation was simulated using a five-parameter model adapted from Connolly and Glaser [4]. The model estimates daily changes in total body concentration by balancing uptake, loss, and growth. Models were run using the R statistical package (R core development team) using the following equation:
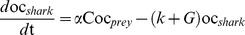
where oc*_shark_* is the concentration of the contaminant in the shark (µg/g (ww)), α is the gross assimilation efficiency (% of ingested mass), C is the prey consumption rate (g(ww)/g(ww)-d), oc*_prey_* is the prey contamination level (µg/g(ww)), *k* is the total contaminant excretion rate (1/d), and G is the growth rate (g(ww)/g(ww)-d). All parameters used in the model can be found in Table 2. The model, incorporated YOY white shark growth rate, daily rationing, absorption efficiency, prey contaminant concentration, and metabolic turnover of contaminants. Consumption rate was based on a 3% of body weight/day derived from captive feeding of YOY white sharks at the Monterey Bay Aquarium, the only approximation for this species [50]. Potential prey contaminant concentrations were obtained from a 2009 health advisory report for southern California fishes [51] to ensure the most up-to-date and potentially most contaminated prey items were used in the model. This model assumes young white sharks are consuming the most heavily contaminated fish prey available in southern California to obtain the maximum levels possible in one year. Metabolic turnover and excretion of contaminants was assumed to be zero in our model to maximize simulated contaminant bioaccumulation. Each iteration of the model simulates accumulation over one day, and the model was run for 365 days to estimate OC bioaccumulation over an entire year. To compare our model levels expressed in total body concentration (µg/g ww total mass) to our observed liver concentrations (µg/g ww liver) a correction factor was used. Sharks sampled had an average hepatosomatic index (HSI) of 12.7±3.1% (n = 12) total body mass. To maximize our simulated concentrations we assumed that 100% of ingested contaminants would fractionate into the liver. Therefore, total values were corrected by multiplying model output by (1 g Total Mass/0.127±0.031 g Liver) to obtain liver concentrations.

**Table 2:** Parameter values used to simulate dietary bioaccumulation over 365 days.

Variable	Parameter	Units	Value	Source
Α	Assimilation efficiency	%	80	Wetherbee and Gruber, 1993
C	Prey consumption rate	g(ww*)/g(ww)-d	3	Ezcurra et al. 2012
OC*_prey_*	Prey Concentration (whole body)	µg/g(ww); ppm	ΣDDT: 0.609 ΣPCB: 0.113	Klasing et al. 2009
G	Growth rate	g(ww)/g(ww)-d	0.3	Ezcurra et al. 2012
*K*	Excretion rate	1/d	0	Present study

* = wet weight.

## Results

**Figure 2:**
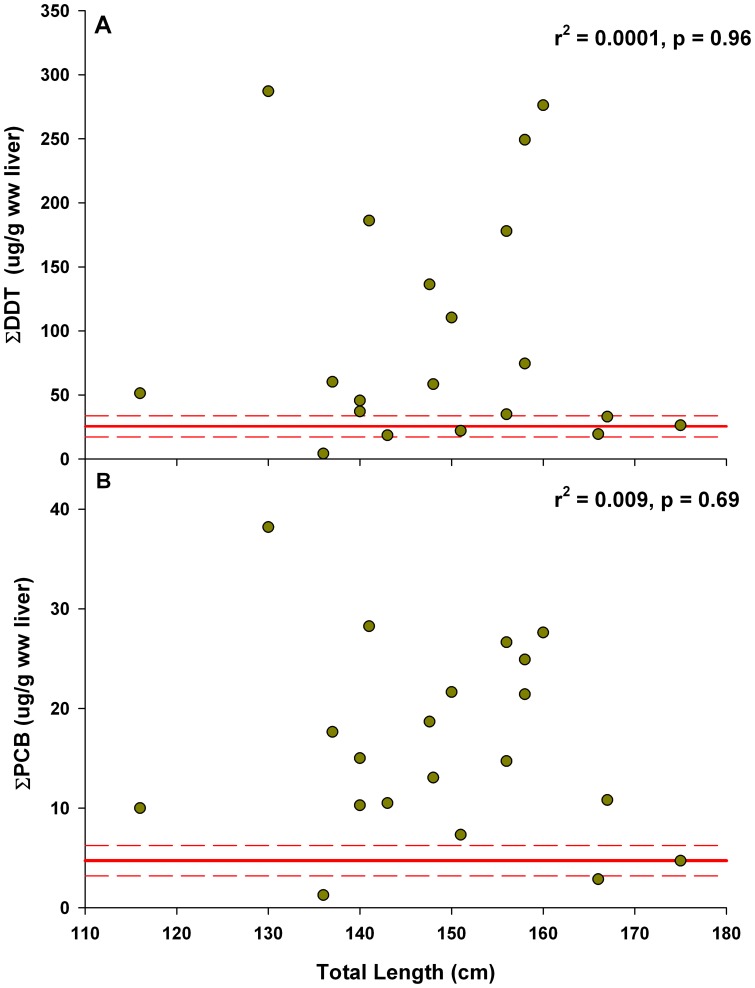
Measured concentration of organochlorine contaminants (µg/g, wet weight) in liver of YOY white sharks across total length (TL, cm), compared with maximum expected dietary accumulation over one year. (A) Dietary accumulation of ΣDDT was estimated to be 25.1±8.23 µg/g (solid and broken reference lines). There was no significant relationship between TL and ΣDDT levels. (B) Dietary accumulation of ΣPCBs was estimated to be 4.73±1.53 µg/g (solid and broken reference lines). There was no significant relationship between TL and ΣPCBs levels.

Organochlorine contaminants levels found in the liver of YOY white sharks were extremely high in comparison to published records for other sharks, particularly in light of their age [16]. Mean (± SD) ∑DDT and ∑PCBs for YOYs were 95.4±91.4 µg/g ww (wet weight) (Figure 1A) and 16.4±9.7 µg/g ww (Figure 1B) (Table 1), respectively. Mean values for all contaminants measured for all YOY white sharks sampled exceeded the maximum simulated levels in the five parameter dietary bioaccumulation model (Figure 2). Running the model for 365 days suggested the potential mean levels that YOY white sharks could attain from dietary exposure are 25.1±8.23 µg/g ∑DDT and 4.73±1.53µg/g ∑PCBs ww in liver (Figure 2). Observed concentrations of both ∑DDT and ∑PCBs in liver of most YOY sharks exceeded maximum model output. ΣDDT concentrations in 16 individuals (80%) exceeded estimated maximum dietary accumulation (Figure 2A), and 17 individuals (85%) exhibited ∑PCBs concentrations exceeding estimated maximum dietary accumulation (Figure 2B). There was no clear relationship between total length and wet weight (µg/g liver weight) (∑DDT: r^2^ = 0.00015, p = 0.96; ∑PCBs: r^2^ = 0.009, p = 0.69) or lipid normalized (µg/g lipid weight) contaminant levels (∑DDT: r^2^ = 0.0014, p = 0.87; ∑PCBs: r^2^ = 0.001, p = 0.89).

**Figure 3:**
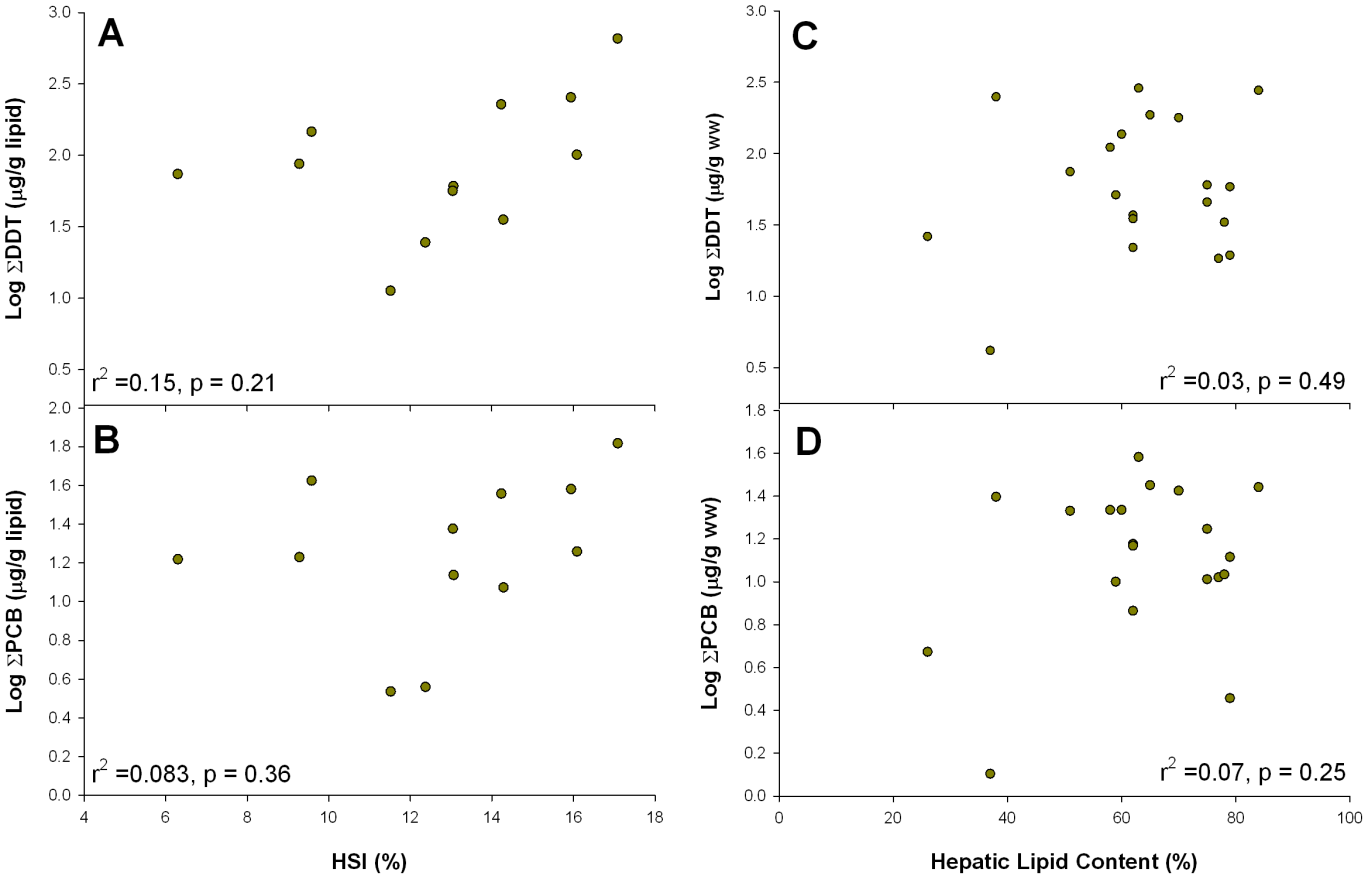
Observed levels of organochlorine contaminants versus metrics of body condition. There was no significant relationship between HSI and ΣDDT (A) or ΣPCBs (B). Hepatic lipid levels were positively related to both ΣDDT (C) and ΣPCBs (D).

The elevated organochlorine concentrations observed in YOY white sharks did not appear to be an artifact of sampling animals in poor condition (i.e. low HSI or hepatic lipid content). There was no significant relationship between ΣDDT and HSI (r^2^ = 0.15, p = 0.21) (Figure 3A), or ΣPCBs and HSI (r^2^ = 0.08, p = 0.36) (Figure 3B). There was no relationship between hepatic lipid content and ∑DDT (r^2^ = 0.03, p = 0.49) (Figure 3C) or ∑PCBs (r^2^ = 0.07, p = 0.25) (Figure 3D).

## Discussion

Observed organochlorine contaminant levels in YOY white sharks were considerably higher than estimated by modeled dietary accumulation alone, with mean liver concentrations of ΣDDT and ΣPCBs approximately 3-fold higher than modeled levels. While sharks were sampled across the entire YOY size range, several were near size at birth, and likely weeks to months old, yet many had contaminant levels exceeding the model, which was run for an entire year. This indicates that mothers are likely passively transferring organochlorine contaminants to embryos during gestation, since it is unlikely that YOY sharks are acquiring such high levels from diet alone. While there was a large variation in observed liver concentrations, only four sharks fell below the modeled dietary accumulation of ΣDDT, and three below modeled dietary accumulation of ΣPCBs. These findings suggest that some, if not all, young white sharks are exposed to some level of organochlorine contaminants *in utero*, although the magnitude of exposure is highly variable.

High levels of organochlorine contaminants are common in marine apex predators, though the magnitude of tissue concentrations seen in YOY white sharks is surprising given their age, and may be due to the feeding ecology of adults. The levels of ΣDDT and ΣPCBs found in sharks in our study are among the highest ever reported for any elasmobranch, despite other studies focusing predominantly on older adults [16]. Though organochlorine contaminant burdens have been examined in other apex-predatory sharks, white sharks are unique in that adults feed heavily on adult pinnipeds during certain times of year [52], [53], while other sharks are predominantly piscivorous. Organochlorine contaminant levels in juvenile orcas (*Orcinus orca*) also vary significantly with adult feeding ecology [54], [55]. One year old piscivorous resident orcas had much lower blubber concentrations of ΣDDT and ΣPCBs, than a one year old transient orca calf, whose mother fed on marine mammals [54]. This lone transient calf had blubber concentrations of 240 µg/g ww ΣDDT and 120 µg/g ww ΣPCBs, which was 3 and 8 fold higher than mean ΣDDT and ΣPCBs in YOY sharks from our study, respectively, though three sharks in our study exceeded this ΣDDT level. The elevated levels of organochlorine contaminants seen in the offspring of marine mammal predators compared with fish predators suggests that maternal prey selection can significantly increase the level of contaminant exposure to offspring during lactation.

The degree and magnitude of contaminant offloading in marine mammals has been linked to the reproductive life-history of mothers [12], in particular birth order of offspring [22], [23], [54], [55], [56], [57]. During their first reproductive event, marine mammal females will offload a significant portion of the contaminants they have acquired prior to maturity, which represents a potentially large reservoir for late maturing apex predators such as orcas [55]. During subsequent reproductive events the reservoir of contaminants stored in lipid reserves will be lower, thus the magnitude of contaminant offloading will decrease. For example, in northern fur seals (*Callorhinus ursinus*) and several species of cetaceans, the greatest difference in the amount of contaminants passed to offspring is between first-time and older mothers [22], [23], [55], [57], [58]. Comparable processes are expected to occur in white sharks given their similarity in life-history characteristics to marine mammals. The individuals observed with relatively high levels of organochlorine contaminants could potentially be the offspring of first-time mothers; however, to directly test this hypothesis we would need to match pups to their mother. The high variability of organochlorine concentrations observed in YOY sharks is likely due to the specific life-history of their mothers, including where females forage geographically and reproductive history. Since observed contaminant concentrations in YOY white sharks were highly variable it suggests that maternal offloading and the processes influencing the magnitude of transfer are important factors related to the level of contaminant exposure sharks will experience as juveniles.

Variability in maternal offloading likely reduces the ability to detect bioaccumulation or growth dilution in young sharks. We saw no significant relationship between ΣDDT and ΣPCBs (wet weight or lipid normalized weight) with total length. While YOY sharks are likely accumulating contaminants feeding in the Southern California Bight, the varying levels of exposure *in utero* and individual growth rates will potentially obscure any trends. Although it is likely that the more heavily chlorinated PCB congeners are accumulated at greater rates, we saw no evidence of accumulation trends across the size range of sharks examined in this study. Future investigations of contaminants in young individuals should focus on the biochemical pathways in which various compounds are transferred between mother and embryo, as modes of maternal supplement during gestation vary among species.

### Conclusions

To accurately gauge the potential impacts of organochlorine contaminants released into the marine environment, we must understand how they cycle through ecosystems, and food webs in particular. The life-history traits (e.g. longevity, low fecundity, apex predatory role) of most sharks make them vulnerable to not only overfishing but to bioaccumulation of contaminants and intergenerational transfer. Though improved fisheries management in southern California has decreased the fishing induced mortality rate of young white sharks [39], the effects of exposure to anthropogenic contaminants is unknown and could affect white sharks throughout their lives. While we did not measure bioindicators of pathology in the current study, there is reason for concern about the long-term physiological and population level consequences of contaminants to white sharks, especially at the early life stages. Many organochlorine contaminants have been well documented to have negative impacts on reproduction and development in a variety of organisms [5], [59], [60], [61]. Unfortunately, no information regarding toxicity of organochlorines exists in sharks, and we have not observed any sub-lethal impacts of these levels to date. Continued monitoring as well as investigating the potential sub-lethal impacts of these contaminants on these and other elasmobranchs may be essential in documenting long term population impacts and contaminant remediation strategies in the future.
